# Epidermal neural crest stem cell transplantation as a promising therapeutic strategy for ischemic stroke

**DOI:** 10.1111/cns.13370

**Published:** 2020-04-12

**Authors:** Mohammad Saied Salehi, Sareh Pandamooz, Anahid Safari, Benjamin Jurek, Amin Tamadon, Mohammad Reza Namavar, Mehdi Dianatpour, Leila Dargahi, Negar Azarpira, Sadegh Fattahi, Seyed Mostafa Shid Moosavi, Somaye Keshavarz, Zahra Khodabandeh, Shahrokh Zare, Somayeh Nazari, Mojdeh Heidari, Sadegh Izadi, Maryam Poursadeghfard, Afshin Borhani‐Haghighi

**Affiliations:** ^1^ Clinical Neurology Research Center Shiraz University of Medical Sciences Shiraz Iran; ^2^ Neuroscience Research Center Shahid Beheshti University of Medical Sciences Tehran Iran; ^3^ Stem cell Technology Research Center Shiraz University of Medical Sciences Shiraz Iran; ^4^ Department of Behavioral and Molecular Neurobiology Faculty of Biology and Preclinical Medicine University of Regensburg Regensburg Germany; ^5^ The Persian Gulf Marine Biotechnology Research Center Bushehr University of Medical Sciences Bushehr Iran; ^6^ Transplant Research Center Shiraz University of Medical Sciences Shiraz Iran; ^7^ Cellular & Molecular Biology Research Center Health Research Institute Babol University of Medical Sciences Babol Iran; ^8^ Department of Physiology School of Medicine Shiraz University of Medical Sciences Shiraz Iran

**Keywords:** bone marrow mesenchymal stem cells, cell therapy, cerebral ischemia, epidermal neural crest stem cells, Infarct volume, neurological deficits

## Abstract

**Introduction:**

Cell‐based therapy is considered as promising strategy to cure stroke. However, employing appropriate type of stem cell to fulfill many therapeutic needs of cerebral ischemia is still challenging. In this regard, the current study was designed to elucidate therapeutic potential of epidermal neural crest stem cells (EPI‐NCSCs) compared to bone marrow mesenchymal stem cells (BM‐MSCs) in rat model of ischemic stroke.

**Methods:**

Ischemic stroke was induced by middle cerebral artery occlusion (MCAO) for 45 minutes. Immediately after reperfusion, EPI‐NCSCs or BM‐MSCs were transplanted *via* intra‐arterial or intravenous route. A test for neurological function was performed before ischemia and 1, 3, and 7 days after MCAO. Also, infarct volume ratio and relative expression of 15 selected target genes were evaluated 7 days after transplantation.

**Results:**

EPI‐NCSCs transplantation (both intra‐arterial and intravenous) and BM‐MSCs transplantation (only intra‐arterial) tended to result in a better functional outcome, compared to the MCAO group; however, this difference was not statistically significant. The infarct volume ratio significantly decreased in NCSC‐intra‐arterial, NCSC‐intravenous and MSC‐intra‐arterial groups compared to the control. EPI‐NCSCs interventions led to higher expression levels of *Bdnf*, *nestin*, *Sox10*, *doublecortin*, *β‐III tubulin*, *Gfap,* and *interleukin‐6*, whereas *neurotrophin‐3* and *interleukin‐10* were decreased. On the other hand, BM‐MSCs therapy resulted in upregulation of *Gdnf*, *β‐III tubulin,* and *Gfap* and down‐regulation of *neurotrophin‐3*, *interleukin‐1,* and *interleukin‐10*.

**Conclusion:**

These findings highlight the therapeutic effects of EPI‐NCSCs transplantation, probably through simultaneous induction of neuronal and glial formation, as well as *Bdnf* over‐expression in a rat model of ischemic stroke.

## 1 INTRODUCTION

Globally, stroke is among the main causes of death and disability. Although thrombolysis and mechanical thrombectomy have revolutionized the treatment of ischemic stroke, the issues of availability, narrow time windows, risk of hemorrhage, and treatment failure are serious drawbacks.^1^, ^2^, ^3^, ^4^ Hence, seeking new alternatives to treat ischemic stroke in order to ameliorate neurological function and reduce mortality is of paramount necessity. Stem cell‐based therapies have the potential to induce angiogenesis, neurogenesis, and synaptic plasticity, and represent a novel and promising regenerative strategy. In this regard, various types of stem cells, including embryonic, neural, induced pluripotent, and mesenchymal stem cells (MSCs), as well as endothelial progenitor and vascular progenitor cells, have been employed and their curative potentials have been evaluated in the treatment of ischemic stroke.^5^ Among them, bone marrow‐derived MSCs (BM‐MSCs) are the most commonly used MSCs, due to their safety, weak immunogenicity, and easy‐to‐culture capabilities.^6^ Several lines of evidence indicate that BM‐MSCs affect the pathological processes underlying ischemic stroke through multiple mechanisms, including inhibition of apoptosis, secreting neurotrophic factors, inducing angiogenesis, and modulating the immune system.^7^ However, bone marrow aspiration is a highly invasive procedure, causing severe pain at the harvesting site. This procedure‐associated pain is considered as one of the major limitations of intraoperative stem cell therapy approaches^8^; hence, alternative sources from which to isolate autologous stem cells should be considered.

Epidermal neural crest stem cells (EPI‐NCSCs) are remnants of the embryonic neural crest, residing in the bulge of adult hair follicle. These cells, similar to their neural crest origin, can be differentiated into various cell types, such as neurons,^9^ glial cells,^10^ osteocytes, and melanocytes.^11^ EPI‐NCSCs were initially introduced in 2004,^12^ with advantages such as high plasticity, abundancy, and accessibility through a minimal invasive procedure, as well as not having ethical issues and graft rejection.^13^ Furthermore, EPI‐NCSCs express a variety of neurotrophic factors, such as brain‐derived neurotrophic factor (BDNF), nerve growth factor (NGF), glial cell‐derived neurotrophic factor (GDNF), neurotrophin‐3 (NT‐3), as well as angiogenic factors such as vascular endothelial growth factor (VEGF) and extracellular proteases that have the capability of supporting cell survival and neo‐vascularization.^14^, ^15^, ^16^, ^17^ In this regard, EPI‐NCSCs might be promising donor cells in the treatment of ischemic stroke, as its beneficial effects have been reported in animals^18^ and ex vivo^19^ models of spinal cord injury, peripheral nerve injury,^13^ as well as Alzheimer's disease.^20^

Intracranial, intra‐arterial, and intravenous application are three effective routes of postcerebral ischemia stem cell administration. Intracranial transplantation is of more invasive nature and can do damage to healthy tissue, as a result of the direct application of cells. Therefore, in the present study, the therapeutic effect of BM‐MSCs and EPI‐NCSCs following intra‐arterial and intravenous administration was compared in a rat model of transient middle cerebral artery occlusion (MCAO). In this regard, neurological function was evaluated before ischemia and 1, 3, and 7 days after transplantation. In addition, infarct volume ratio and relative expression of 15 selected target genes in three categories of trophic factors, cellular markers, and inflammatory cytokines were evaluated 7 days after cell therapy.

## 2 MATERIAL AND METHODS

### 2.1 Animals and ethics statement

In the present study, 82 Sprague Dawley male rats weighing 240‐260 g at the beginning of the experiment were used. All rats were housed under controlled conditions and allowed ad libitum access to standard food and water. This experiment was approved by the Animal Care Committee of Shiraz University of Medical Sciences, Shiraz, Iran, and was carried out in compliance with the recommendations of the Care and Use of Laboratory Animals (National Academy Press, 1996, Washington, USA).

### 2.2 Experimental groups

Experimental animals were randomly divided into 6 groups: (a) sham (n = 12), receiving surgical procedures similar to other groups without middle cerebral artery occlusion and cell transplantation; (b) control or MCAO (n = 12), underwent 45 minutes MCAO and received 0.5 mL phosphate‐buffered saline (PBS); (c) NCSC‐IA (n = 12), subjected to 45 minutes MCAO and received intra‐arterially administered EPI‐NCSCs; (d) NCSC‐IV (n = 12), experienced 45 minutes MCAO and EPI‐NCSCs were applied intravenously; (e) MSC‐IA (n = 12), underwent 45 minutes MCAO and received intra‐arterially administered BM‐MSCs, and (f) MSC‐IV (n = 12), subjected to 45 minutes MCAO and BM‐MSCs were injected intravenously.

### 2.3 Stem cell preparation

To isolate EPI‐NCSCs, rat whiskers pad (n = 10) were micro‐dissected to obtain individual hair follicles. After several washes, the capsule of follicles was cut longitudinally and the bulge region within the follicles rolled out. Bulges of hair follicles were explanted on collagen‐coated 12‐well cell culture plates and fed with minimum essential medium‐α (α‐MEM, Sigma‐Aldrich) contained 5% day‐11 chick embryo extract, 10% fetal bovine serum (FBS, Gibco), and 1% penicillin/streptomycin (P/S, Gibco) and were incubated in a humidified atmosphere at 37°C with 5% CO_2_. Half of the culture medium was renewed every day, and 7 days after stem cell migration, cells were detached using 0.25% trypsin/EDTA (Gibco) and passaged. This procedure was described in detail in previous publications.^21^, ^22^

Verified rat BM‐MSCs were purchased from the Iranian Biological Resource Center (Tehran, Iran) and expanded in high glucose Dulbecco's modified Eagle's medium (DMEM‐HG) supplemented with 10% FBS and 1% P/S. Both BM‐MSCs and EPI‐NCSCs were isolated from 10‐ to 14‐week old male Sprague Dawley rats and harvested at passage number 4‐6 for grafting.

### 2.4 EPI‐NCSCs verification

Immunostaining against neural crest stem cell marker (nestin), neural crest cells marker (SOX10), immature neurons markers (doublecortin and β‐III tubulin), and glial marker (GFAP) were performed to verify expanded EPI‐NCSCs. Briefly, cultured EPI‐NCSCs were fixed with 4% paraformaldehyde and washed with PBS containing 0.05% Tween‐20. Then, cells were blocked with 1% bovine serum albumin containing 0.2% triton X‐100 and incubated overnight at 4°C with primary antibodies: rabbit anti‐nestin, anti‐SOX10, anti‐doublecortin, anti‐β‐III tubulin and anti‐GFAP (#ab93157, ab155279, ab77450, ab18207, and ab7260; Abcam). Following washing with PBS, cells were incubated with FITC‐conjugated secondary antibody (Sigma, #F1262) and counterstained with DAPI (Sigma, #D9564). Images were captured with the Olympus inverted fluorescence microscope.

### 2.5 MCAO procedure

Experimental rats were subjected to transient MCAO as it was described earlier.^23^ In brief, animals were anesthetized with chloral hydrate (320 mg/kg, intraperitoneally) and following midline incision of the neck, right common carotid artery, right external carotid artery, and right pterygopalatine artery were ligated. A silicone rubber‐coated monofilament (#403556, Doccol Corporation) was inserted into the right common carotid artery and advanced cranially to the internal carotid artery until a mild resistance felt in order to occlude the blood flow of the right middle cerebral artery. For reperfusion, the filament was carefully removed after 45 minutes. Laser Doppler was used to monitor microvascular blood flow reduction during the surgery. Also, during the surgical procedure, rectal temperature was monitored and maintained at 37°C using a heating lamp and heating pad.

### 2.6 Transplantation approaches

In the intra‐arterial groups, immediately after removing the suture, the common carotid artery ipsilateral to the MCAO was cannulated using PE 20 (Clay Adams lnc.), and 2 × 10^6^ cells (BM‐MSCs or EPI‐NCSCs) in 0.5 mL PBS were injected directly into the artery over the course of 1 minute. For intravenous delivery, 2 × 10^6^ cells were suspended in 0.5 mL PBS and injected into the tail vein immediately after suture withdrawal.

### 2.7 Behavioral test

In all experimental groups, the behavioral test was performed before ischemia (day 0) and 1, 3 and 7 days after the surgery/stem cell transplantation. In doing so, neurological function was graded on a scale of 0‐4 as follows, 0: no neurological deficit; 1: unable to fully extend left forepaw (mild); 2: leftward circling (moderate); 3: falling to the left (severe); and 4: minimal level of consciousness without spontaneous walking.^24^ In all experimental groups, MCAO rats with scores 3 or 4 died within 2‐7 days after the ischemia and were excluded from the experiment. Furthermore, MCAO rats with score 0 at day 1 were also excluded from the experiment.

### 2.8 Measurement of infarct volume ratio by TTC (2,3,5‐triphenyltetrazolium chloride) staining

Seven days after the surgery/stem cell transplantation, half of the animals in each experimental group were subjected to quantification of infarct volume ratio. Under deep anesthesia, rats were killed, brains were removed quickly and coronal sections with 2 mm thickness prepared. Then, brain sections were incubated for 30 minutes at 37°C in 1% TTC (Sigma) and infarct volume ratio was evaluated using ImageJ software.

### 2.9 Evaluation of the target genes using qRT‐PCR

Seven days after the surgery/stem cell transplantation, the other half of animals in each experimental group were killed under deep anesthesia, brains were removed immediately, and the striatum as well as the cortex ipsilateral to the MCAO were dissected, snap‐frozen, and stored at −80°C until further processing. Total RNA extraction (YTzol Pure RNA buffer, Yekta Tajhiz Azma), DNase treatment (Thermo Scientific), and cDNA synthesis (cDNA Synthesis kit, Yekta Tajhiz Azma) were performed on the striatum and cortex samples based on the manufacturer's instructions.

In the present study, relative expression of 15 genes in three categories was evaluated as follows: **1**‐trophic factors including BDNF, NGF, GDNF, NT‐3, and VEGF; **2**‐cellular markers including nestin, SOX10, doublecortin (DCX), β‐III tubulin, GFAP, β‐actin, and **3**‐inflammatory cytokines including tumor necrosis factor‐α (TNFα), interleukin (IL)‐1β, IL‐6, and IL‐10. To evaluate target genes, qRT‐PCR was performed using first‐strand cDNA template, specific primer sets (presented in Table 1), and SYBR green Master Mix (RealQ Plus 2X, Ampliqon). All samples were run in triplicate. Amplification conditions included 95°C for 15 minutes, and then, 40 cycles of 95°C for 20 seconds and 60°C for 1 minutes were performed on the Applied Biosystems StepOnePlus (ABI, USA). Melting curve analysis revealed just one amplification peak for each reaction, while nontemplate as well as minus reverse transcriptase controls confirmed the absence of genomic contamination. The Ct value for each target gene was normalized to the Ct of hypoxanthine phosphoribosyltransferase‐1 (HPRT1) transcript as a suitable housekeeping gene, according to the previous reports in the in vivo model of MCAO.^25^, ^26^ In addition, 5 μL of amplified products was subjected to electrophoresis on a 1% agarose gel to observe a single band of the expected size. The arithmetic formula 2^−ΔΔCT^ was used to calculate fold changes.^27^

**TABLE 1:** Primer sequences (5′–3′) used in quantitative polymerase chain reaction (qPCR)

Gene	Sequence	Amplicon (bp)
*Ngf*	F‐ CCCAATAAAGGCTTTGCCAAGGAC	78
	R‐ GAACAACATGGACATTACGCTATGC	
*Nt‐3*	F‐ GACACAGAACTACTACGGCAACAG	184
	R‐ ACTCTCCTCGGTGACTCTTATGC	
*Bdnf*	F‐ CGATTAGGTGGCTTCATAGGAGAC	182
	R‐ CAGAACAGAACAGAACAGAACAGG	
*Gdnf*	F‐ GCTGACCAGTGACTCCAATATGC	192
	R‐ CCTCTGCGACCTTTCCCTCTG	
*Vegf*	F‐ ACTTGAGTTGGGAGGAGGATGTC	183
	R‐ GGATGGGTTTGTCGTGTTTCTGG	
*Nestin*	F‐ CAAGGTCTGGTCTGGTGTATGC	106
	R‐ GCTTTATTCAGGGAGGAAGAGAGG	
*Sox10*	F‐ ACGCAGAAAGTTAGCCGACCAG	92
	R‐ CACTCTCGTTCAGCAACCTCCAG	
*Dcx*	F‐ CGCCGCAGCAAGTCTCCAG	185
	R‐ TCGCCAAGTGAATCAGAGTCATCC	
*β‐III tubulin*	F‐ GCTGGAACGCATCAGTGTCTAC	162
	R‐ GCACCACTCTGACCGAAGATAAAG	
*Gfap*	F‐ GGGACAATCTCACACAGGACCTC	162
	R‐ CCTCCAGCGACTCAACCTTCC	
*Actin, Beta*	F‐TCTATCCTGGCCTCACTGTC	122
	R‐AACGCAGCTCAGTAACAGTCC	
*Tnf‐α*	F‐ CCACCACGCTCTTCTGTCTACTG	149
	R‐ GGCTACGGGCTTGTCACTCG	
*Il‐1β*	F‐ TTCAAATCTCACAGCAGCATCTCG	198
	R‐ ACACTAGCAGGTCGTCATCATCC	
*Il‐6*	F‐ AGCCAGAGTCATTCAGAGCAATAC	161
	R‐ GTTGGATGGTCTTGGTCCTTAGC	
*Il‐10*	F‐ CACCCACTTCCCAGTCAGC	148
	R‐ ACCCAAGTAACCCTTAAAGTCCTG	
*Hprt*	F‐ CCAGCGTCGTGATTAGTGATGATG	135
	R‐ GAGCAAGTCTTTCAGTCCTGTCC	

### 2.10 Statistical analysis

SPSS (Version 20) statistical software package (IBM Company) and GraphPad Prism (Version 7.03, GraphPad Software, Inc) were used for statistical analysis and graphing the data. For continuous variables, data are presented as mean ± SEM. Data of the behavioral test were statistically analyzed using the nonparametric Kruskal‐Wallis test. Infarct volume ratio and relative expression of target genes were subjected to the Shapiro‐Wilk normality test and comparisons between groups were made by one‐way ANOVA followed by post hoc Tukey test. *P* < .05 was considered as statistically significant.

## 3 RESULTS

### 3.1 Verification of EPI‐NCSCs

**FIGURE 1:**
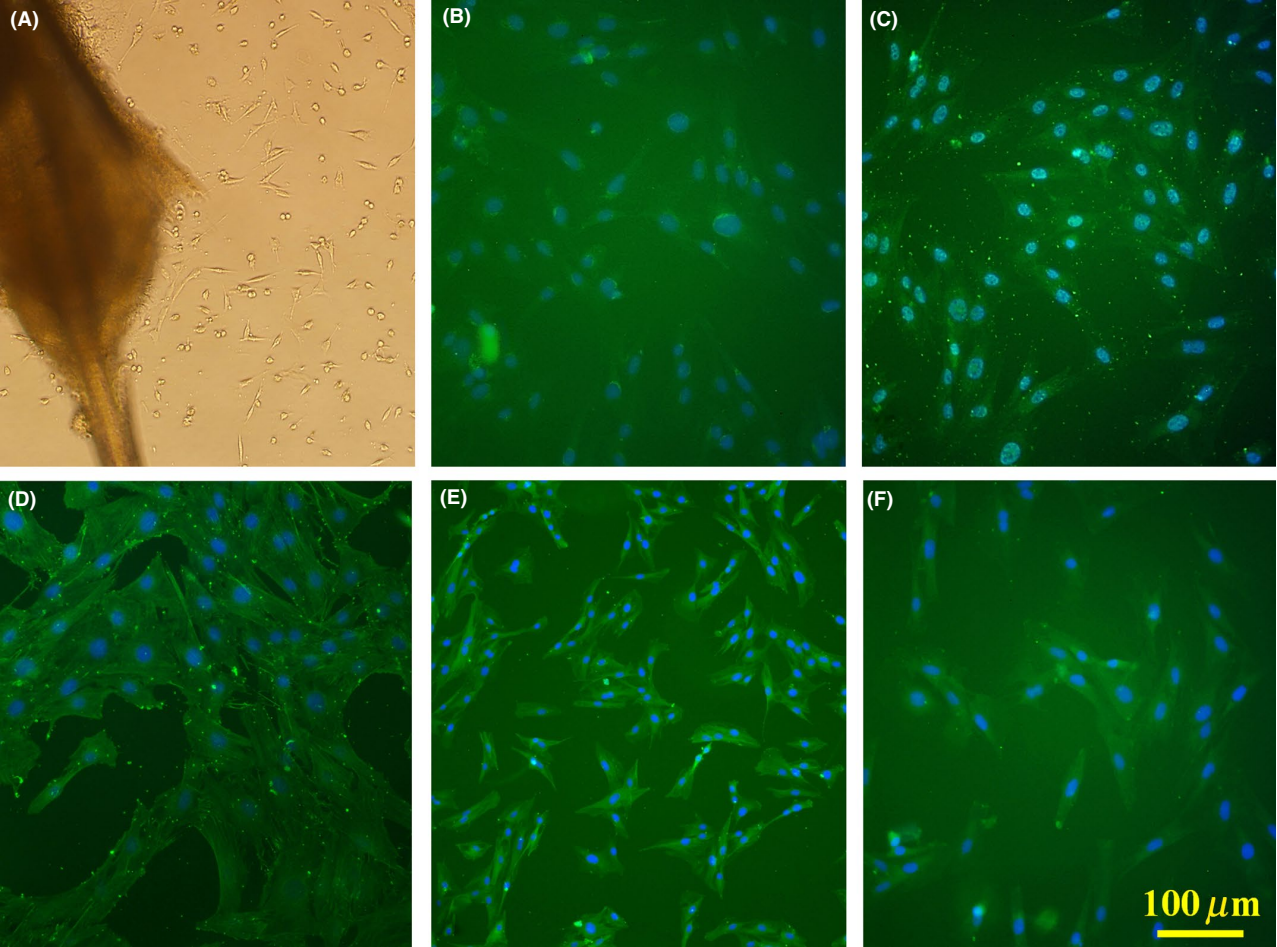
Migrated cells around the bulge, 7 d after explantation (A). Immunostaining against nestin (B), SOX10 (C), doublecortin (D), β‐III tubulin (E), and glial fibrillary acidic protein (F) to verify migrated cells. Cell nuclei counterstained with DAPI. Scale bar: 100 μm

In the current investigation, 2‐3 days after explantation, the migrated cells were observed around the bulges, which increased over time. Immunostaining against nestin, SOX10, DCX, β‐III tubulin, and GFAP revealed the expression of these markers that verified the type of migrated cells as EPI‐NCSCs (Figure 1).

### 3.2 Functional deficits

**FIGURE 2:**
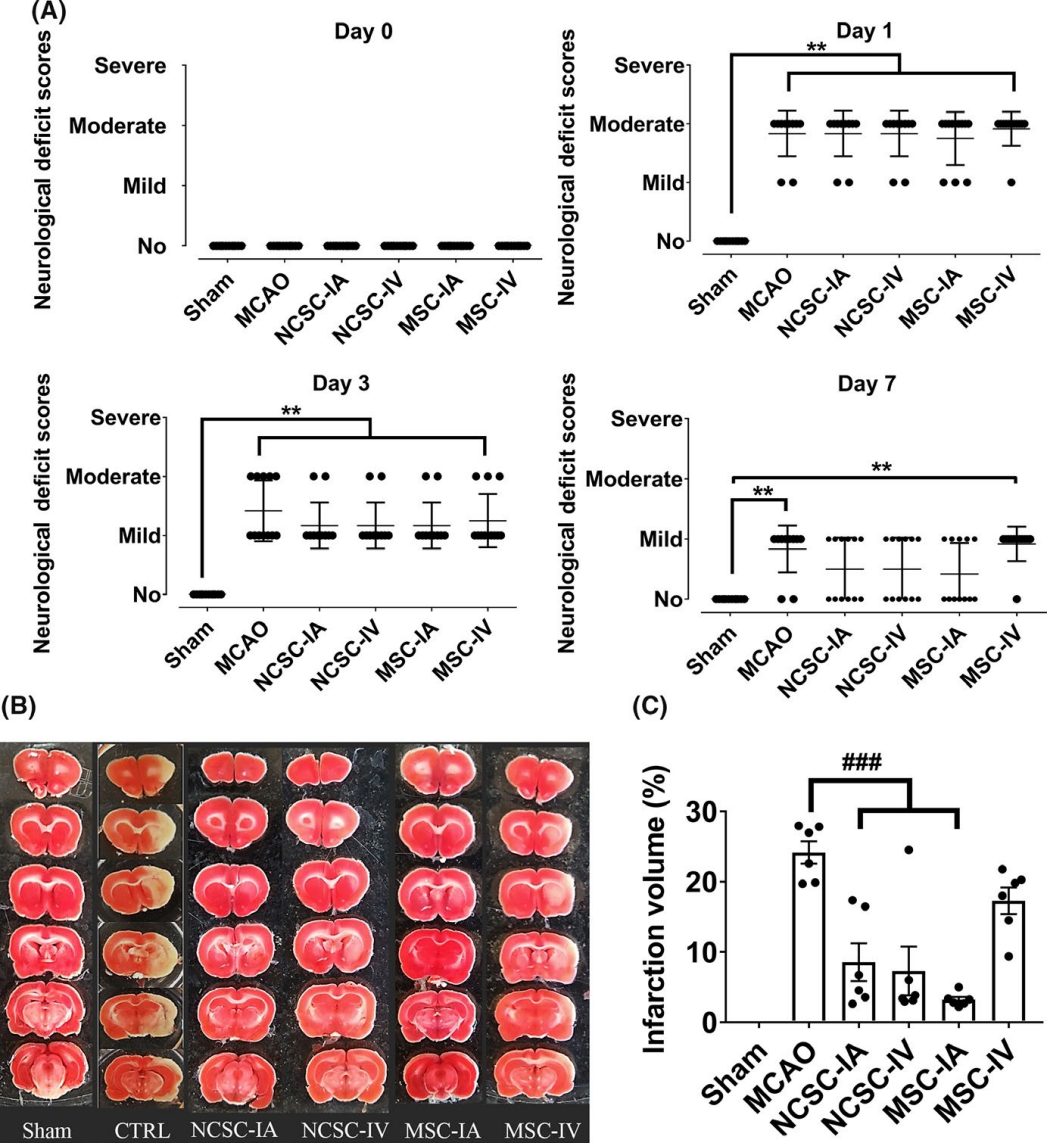
A, Neurological deficit before surgeries (day 0) and 1, 3, and 7 d postischemia/cell therapy. ^**^ *P *< .01 (n = 12 in each experimental group); B, Representative photographs of coronal brain sections 7 days postischemia/cell therapy in six experimental groups stained with 2,3,5‐triphenyltetrazolium chloride and C, Bar graph showing %infarct volumes in each group. ^###^ *P *< .001 (only significant differences compared to MCAO group are pointed; n = 6 in each experimental group)

In the present study, neurological function was assessed before surgeries (day 0) and 1, 3, and 7 days postischemia/cell therapy. Before surgery, no deficits were observed in the experimental groups. At 1 and 3 days postsurgery, the MCAO group as well as all the stem cell transplantation groups exhibited significant functional deficits in comparison to the sham group. Seven days after transplantation, the intra‐arterial administrations of EPI‐NCSCs and BM‐MSCs as well as intravenous administration of EPI‐NCSCs led to a better functional outcome compared to the MCAO group; however, the differences were not statistically significant (Figure 2A).

### 3.3 Infarct volume ratio

Seven days after surgery/stem cell transplantation, the infarct volume ratio was assessed by TTC staining (Figure 2B). Here, the ipsilateral hemisphere was severely damaged in the MCAO group (24 ± 1.6% of the brain volume). The infarct volume ratio had significantly decreased in NCSC‐IA (8.6 ± 2.7%), NCSC‐IV (6.8 ± 3.5%) and MSC‐IA (2.9 ± 0.22%) groups, but not in the MSC‐IV (17 ± 2.3%) group, compared to the control (Figure 2C).

### 3.4 Relative expression of target genes

Seven days after surgery/stem cell transplantation, relative expression of 15 genes in three categories of trophic factors, cellular markers, and inflammatory cytokines was evaluated in the striatum as well as cortex of all the experimental groups.

**FIGURE 3:**
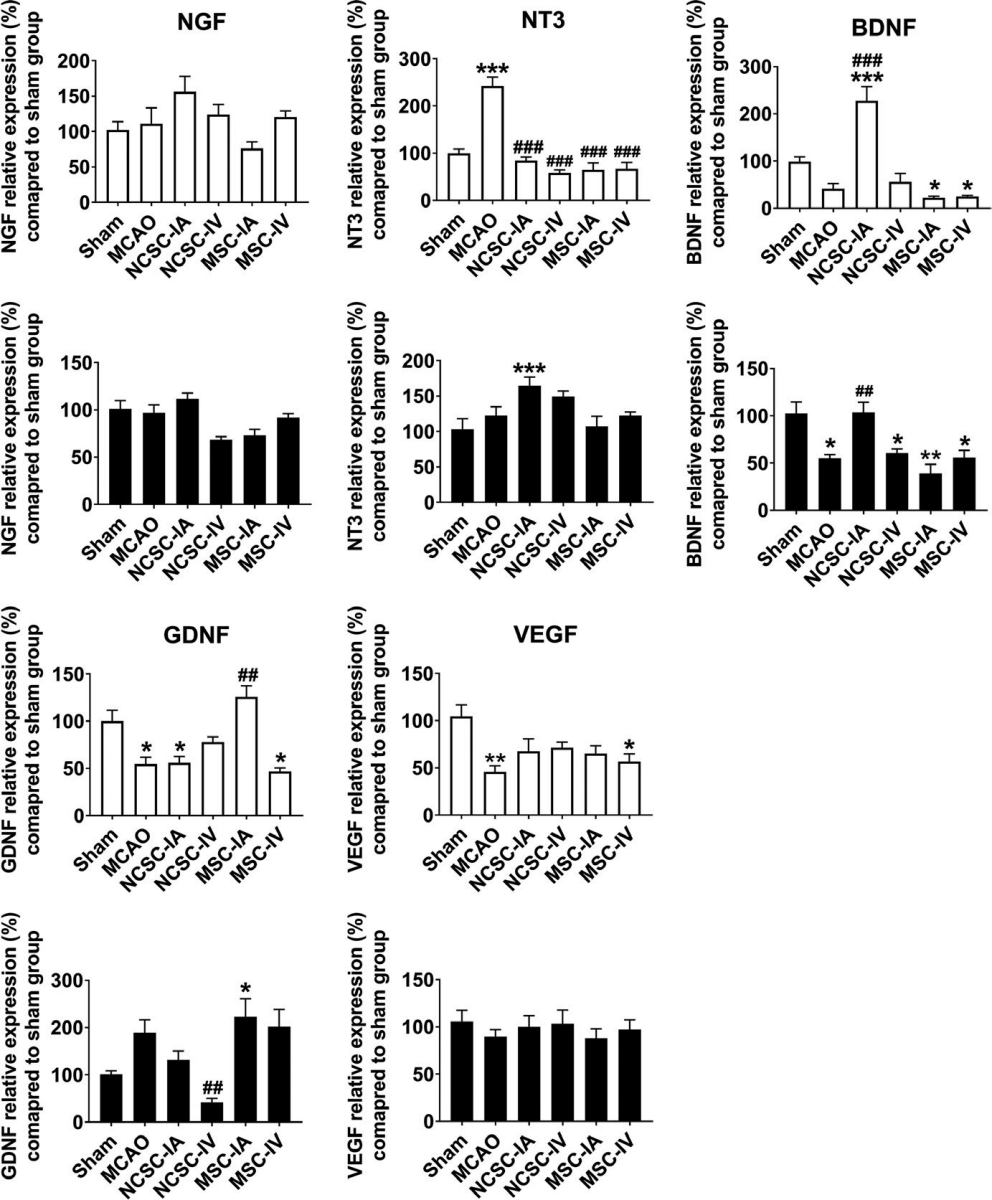
Relative expression of nerve growth factor (NGF), neurotrophin‐3 (NT‐3), brain‐derived neurotrophic factor (BDNF), glial cell‐derived neurotrophic factor (GDNF), and vascular endothelial growth factor (VEGF) 7 d postischemia/cell therapy in the striatum as well as cortex of six experimental groups. ^*^ *P *< .05, ^**^ *P *< .01, ^***^ *P *< .001 significant differences compared to sham group; ^##^ *P *< .01, ^###^ *P *< .001 significant differences compared to MCAO group (n = 6 in each experimental group)

In the trophic factors category, relative expression of *Bdnf*, *Gdnf,* and *Vegf* in the striatum region of the MCAO group showed a significant down‐regulation compared with the sham group. In addition, relative expression of *Nt‐3* was upregulated, while the expression of *Ngf* remained unchanged in the MCAO group compared to sham. NCSC‐IA increased *Bdnf* expression whereas MSC‐IA upregulated the *Gdnf* transcript. Both types of stem cells *via* both routes reduced *Nt‐3* mRNAs. In the cortex, *Bdnf* was the only gene that was affected by ischemia and NCSC‐IA elevated its expression (Figure 3).

In the cellular markers category, *Nestin* and *β‐actin* expressions were significantly increased, *β‐III tubulin* decreased, and *Sox10* and *Dcx* expressions remained unchanged in the striatum region of the MCAO group compared to sham. In addition, *Gfap* mRNA had increased more than 500% following ischemia, which failed to reach significance in a one‐way ANOVA due to the number of groups compared; however, independent statistical comparison between the ischemic and control group revealed a significant difference.

**FIGURE 4:**
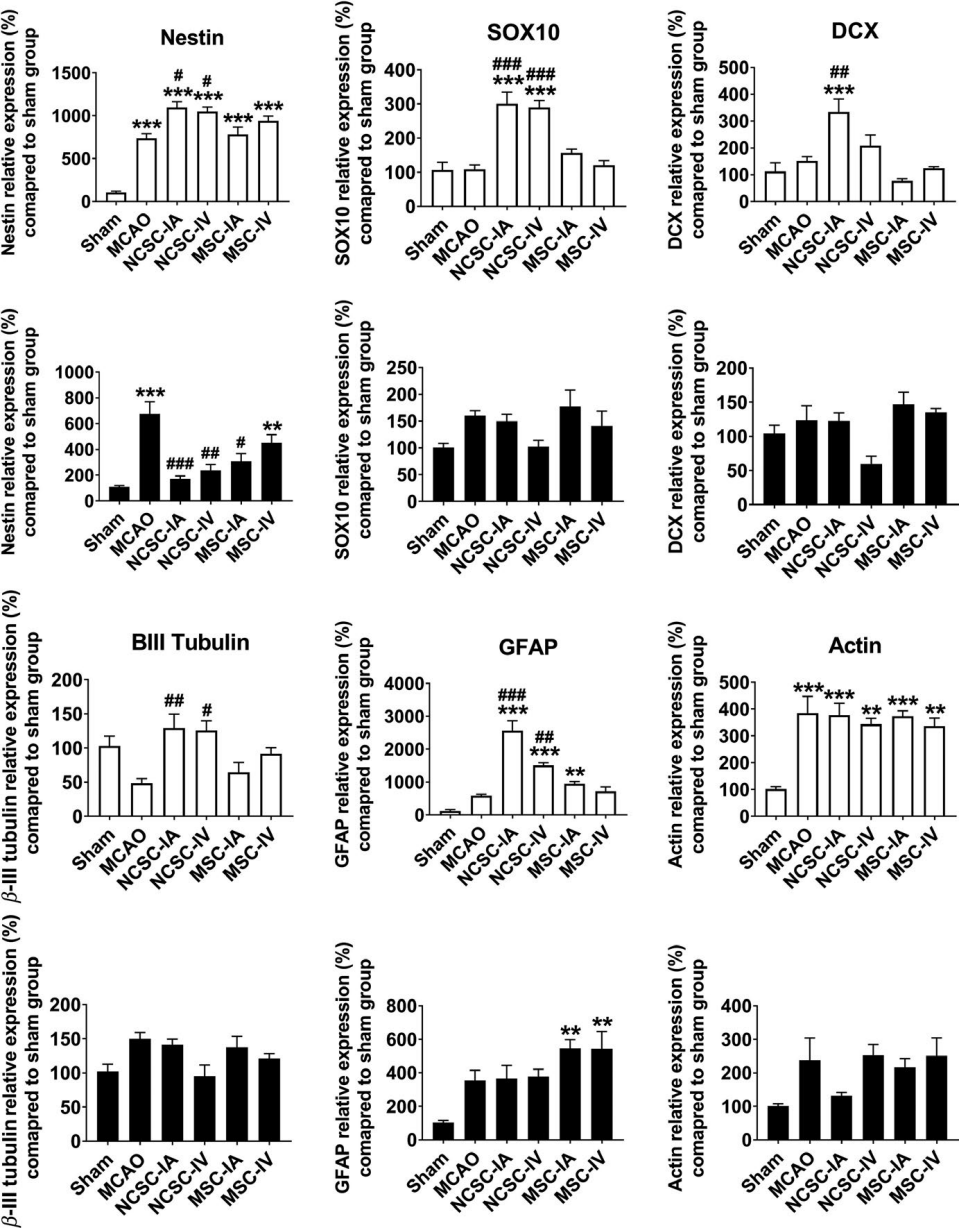
Relative expression of nestin, SOX10, doublecortin (DCX), β‐III tubulin, glial fibrillary acidic protein (GFAP), and β‐actin 7 d postischemia/cell therapy in the striatum as well as cortex of six experimental groups. ^**^ *P *< .01, ^***^ *P *< .001 significant differences compared to sham group; ^#^ *P *< .05, ^##^ *P *< .01, ^###^ *P *< .001 significant differences compared to MCAO group (n = 6 in each experimental group)

EPI‐NCSCs transplantation *via* both routes led to higher expression levels of *Nestin*, *Sox10*, *Dcx*, *β‐III tubulin,* and *Gfap* transcripts. In the cortex, *Nestin* was the only gene that was affected by MCAO and stem cell administration reduced its expression. Again, *Gfap* transcript was upregulated more than 300% following ischemia, which failed to reach statistical significance in a one‐way ANOVA, but was significant after independent statistical comparison; however, BM‐MSCs transplantation led to higher expression levels of *Gfap*, compared to sham (Figure 4).

**FIGURE 5:**
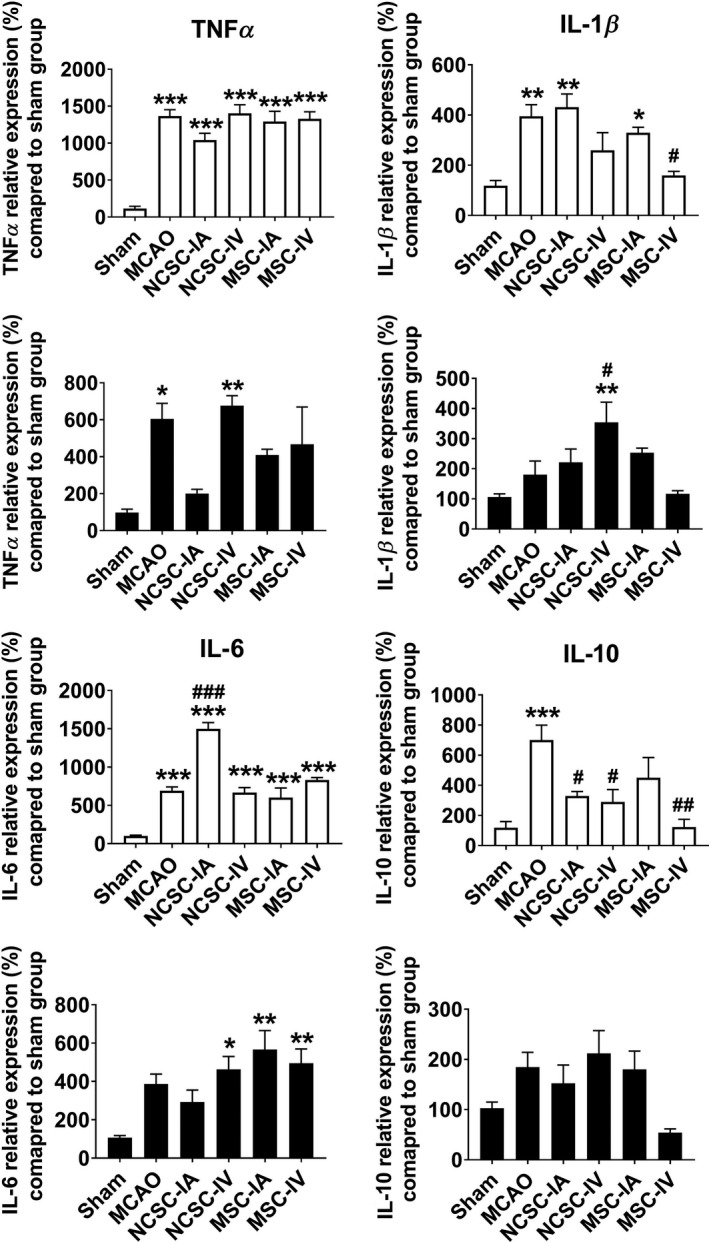
Relative expression of tumor necrosis factor‐α (TNFα), interleukin (IL)‐1β, IL‐6, and IL‐10 7 d postischemia/cell therapy in the striatum as well as cortex of six experimental groups. ^*^ *P *< .05, ^**^ *P *< .01, ^***^ *P *< .001 significant differences compared to sham group; ^#^ *P *< .05, ^##^ *P *< .01, ^###^ *P *< .001 significant differences compared to MCAO group (n = 6 in each experimental group)

**FIGURE 6:**
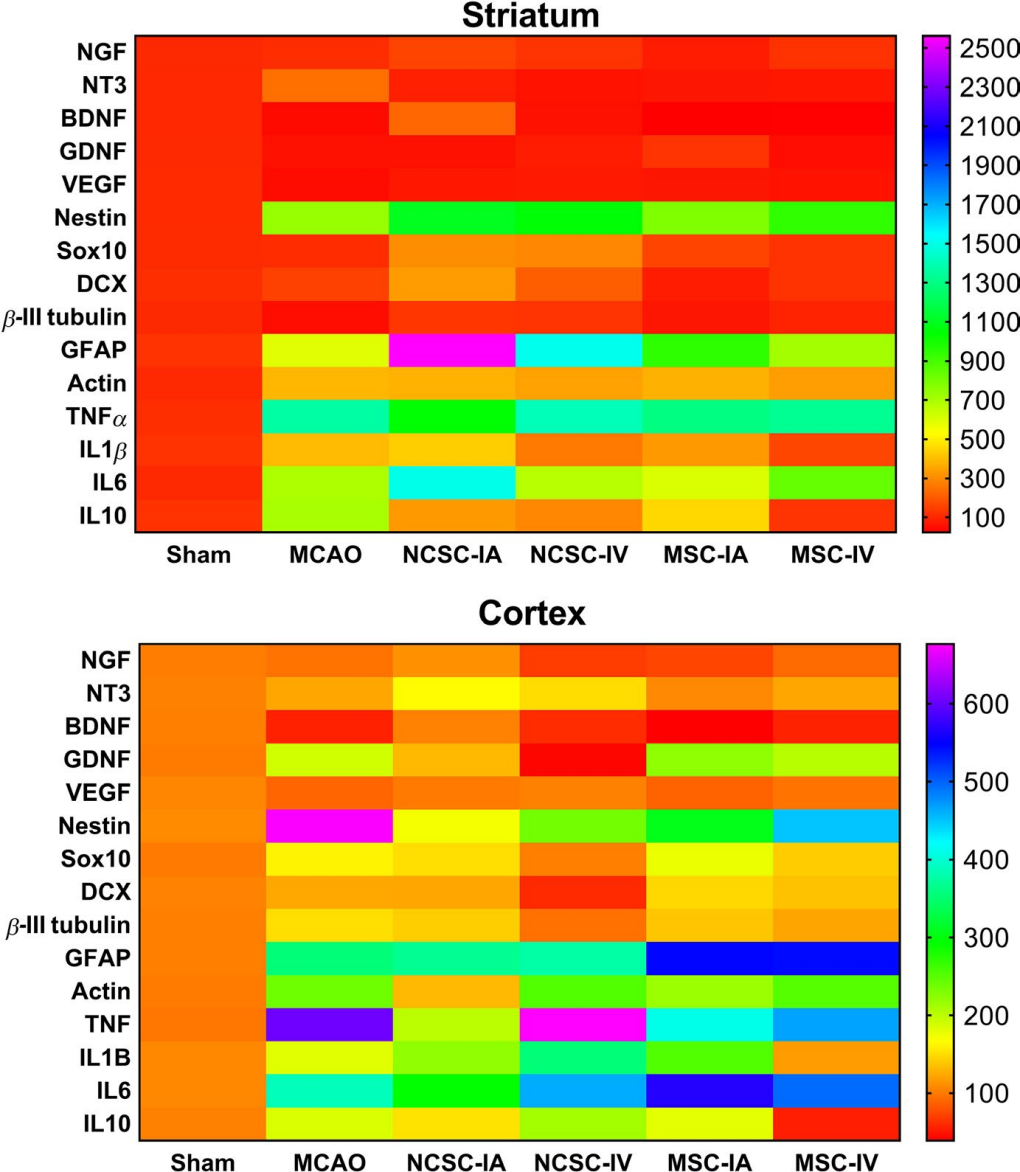
Heat map representation of all evaluated target genes expression in the striatum as well as cortex

In the inflammatory cytokines category, expression of all target genes including *Tnfα*, *Il‐1β*, *Il‐6,* and *Il‐10* was elevated in the striatum region of the MCAO group compared to sham. NCSC‐IA induced the expression of *Il‐6* mRNA, MSC‐IV decreased *Il‐1β* level, and stem cell transplantation reduced *Il‐10* transcripts. In the cortex, *Tnfα* was the only transcript that was statistically affected by MCAO (Figure 5). A heat map representation of all evaluated target genes expression is illustrated in Figure 6.

## 4 DISCUSSION

Stem cell transplantation has been proposed as a promising strategy for stroke patients who do not respond to therapeutic alternatives. In the present study, therapeutic effects of EPI‐NCSCs were assessed in comparison to BM‐MSCs as one the most effective stem cell sources in the rat model of ischemic stroke. In doing so, experimental animals were subjected to 45 minutes MCAO, and immediately following reperfusion, EPI‐NCSCs or BM‐MSCs were transplanted *via* the IA or IV route. Since the optimal time point for EPI‐NCSCs transplantation is unknown, assuming that sooner is better,^28^, ^29^, ^30^ we immediately transplanted both types of stem cells after reperfusion. Also, due to the wide distribution of transplanted stem cells through intravascular approach which might be better for large‐area brain damage,^31^ we administered both types of stem cells via IA as well as IV routes. There is no doubt that IV administration is less invasive and relatively simple; however, small numbers of cells reach the ischemic area. Through IA transplantation, cells are delivered to the injured area in a short time and trapping in other tissues, such as lung tissue, diminishes; however, its effectiveness and safety are debatable.^32^, ^33^, ^34^

In the present investigation, neurological deficits were assessed at different time points. On the 7th day after cell transplantation, we could show that NCSC‐IA, NCSC‐IV, and MSC‐IA led to better nonsignificant functional outcome compared to the MCAO group. Here, althought we did not find any beneficial effects of MSC‐IV on the functional recovery, previous experiments reported the effectveness of MSC‐IV at different time points. Supplementary Tables 1 and 2 summerized some of these reports. On the other hand, our findings clearly exhibited that NCSC‐IA, NCSC‐IV, and MSC‐IA reduced infarct volume ratio compared to the MSC‐IV or MCAO groups. The dichotomy between our pathological and functional outcomes after cell therapy might be dependent on multiple variables such as time of MCAO, type of stem cell, number of employed cell, route of administration, time of transplantation after cerebral ischemia, and eventually time as well as methods of measuring infarct volume and behavioral deficits. This paradigm of pathological improvement without functional outcomes has also been reported in drug‐based therapy of cerebral ischemia.^35^

Striatum and neocortex are two main brain regions that always affected by mild (30 minutes) MCAO.^36^ Hence, we evaluated the relative expression of 15 selective target genes in the striatum as well as cortex 7 days after transplantation. In the striatum, we have shown that relative expression of *Nt‐3*, *Nestin*, *β‐actin*, *Tnfα*, *Il‐1β*, *Il‐6*, and *Il‐10* increased whereas expression of *Bdnf*, *Gdnf*, *Vegf*, and *β‐III tubulin* decreased after cerebral ischemia. *Ngf*, *Dcx*, *Sox10*, and *Gfap* transcripts were not statistically affected by MCAO. *Bdnf*, *Nestin*, and *Tnfα* were the only transcripts, which statistically affected by ischemia in the cortex. EPI‐NCSCs interventions led to greater expression of *Bdnf*, *Nestin*, *Sox10*, *Dcx*, *β‐III tubulin*, *Gfap*, and *Il‐6* while decreased *Nt‐3* and *Il‐10*. On the other hand, BM‐MSCs therapy upregulated the expression of *Gdnf*, *β‐III tubulin*, and *Gfap* whereas down‐regulated *Nt‐3*, *Il‐1*, and *Il‐10* mRNAs. Notably, for both type of stem cells, the most significant differences were obtained by intra‐arterial transplantation in the striatum region.

Releasing trophic factors is an important aspect of cell‐based therapy. Here, we have shown that IA transplantation of EPI‐NCSCs significantly upregulated *Bdnf* mRNA in the ipsilateral hemisphere; however, such effect was not observed following NCSC‐IV or BM‐MSCs administrations. Earlier, it was reported that genetic manipulation of BM‐MSCs that led to over‐expression of BDNF ameliorate the devastating conditions of cerebral ischemia.^37^, ^38^ Also IV injection of BDNF before focal cerebral ischemia^39^ or intraparenchymal administration of BDNF after permanent MCAO^40^ resulted in reduced infarct area. EPI‐NCSCs genuinely express high level of BDNF^16^, ^18^ and IA route grafting probably facilitated access of greater numbers of cells to the damaged site. Therefore, these stem cells can be responsible for enhanced *Bdnf* expression that might be involved in the therapeutic actions of EPI‐NCSCs. The induction of BDNF after this type of stem cell transplantation was also demonstrated in slice culture of spinal cord injury.^19^

Nestin is known as a neuronal progenitor cell marker in the adult brain, and it is well established that nestin‐positive cells can ultimately differentiate into a variety of CNS cell types, including oligodendrocytes, astrocytes, and neurons.^41^, ^42^ Increased nestin expression following ischemic stroke was reported in several investigations, and it was suggested that nestin‐positive cells induced by MCAO eventually shifted toward reactive astrocytes.^43^, ^44^ In line with these reports, our results revealed the upregulation of *Nestin* and *Gfap* (as glial marker) after ischemia. This finding was highlighted in the striatum region after EPI‐NCSCs transplantation, which might be one of the approaches that these stem cells employed to protect injured sites. In this regard, we showed previously that EPI‐NCSCs grafting led to over‐expression of GFAP in an ex vivo model of spinal cord injury, which ultimately ameliorated the devastating condition of damaged tissue.^19^

Furthermore, it was suggested that SOX10 plays a crucial role to direct the fate of neural precursor cells toward the oligodendrocyte lineage.^45^ Here, although cerebral ischemia did not affect *Sox10* expression, EPI‐NCSCs transplantation caused elevated expression of this transcript suggesting formation of different glial cells types.

DCX is widely considered as a marker of neurogenesis as well as neuronal precursor cells. A positive correlation between DCX expression and the extent of adult neurogenesis has been demonstrated previously.^46^, ^47^ Moreover, it was reported that DCX expression in the lesioned brain area following stroke correlates with the recovery of functional deficits.^48^ Transgenic ablation of DCX resulted in exacerbation of stroke outcome and attenuation motor function recovery.^49^, ^50^ Interestingly, our findings of upregulated *Dcx* expression in the brain following EPI‐NCSCs transplantation can be due to the expression of this marker by EPI‐NCSCs and/or enhanced levels of endogenous DCX expression as a result of stem cell grafting. Here, both scenarios might eventually improve the function of the injured area. Remarkably, in the current investigation, we have found that *β‐III tubulin* (as an immature neuronal marker), *Dcx*, *Nestin*, *Gfap*, and *Sox10* are upregulated in the EPI‐NCSCs transplanted groups, suggesting the simultaneous induction of neuronal and glial formation. Lastly, although increased expression of inflammatory cytokines after ischemia was expected,^51^ the pathways through which stem cells modulate inflammatory processes following stroke require further investigation.^52^

One of the main issues in cell transplantation for cerebral ischemia is the selection of suitable types of stem cells. It has been proposed that the ideal cell should have the ability to proliferate and expand ex vivo from the minimal numbers of donor cells. Also, cell transplants should be phenotypically plastic, bear minimal risk of rejection, be free of ethical controversies, and possess the ability to differentiate into appropriate neural and glial cells.^53^ In this regard, EPI‐NCSCs offer several advantages: They possess a high degree of plasticity, generate all major neural crest derivatives, can be isolated as a highly pure population, are abundant and easily accessible, can be expanded in vitro into millions of cells, do not raise ethical concerns, and last but not least show absence of tumorigenicity.^14^, ^21^, ^54^

It is important to note that stroke mostly occurs in elderly people, and it is known that BM‐MSC populations, as well as their differentiation and/or proliferation capacity, dramatically decline with age.^6^, ^55^ To the contrary, recent reports revealed that NCSCs from human epidermis of aged donors maintain their multipotency in vitro and in vivo*.* ^56^ Therefor, EPI‐NCSCs, due to abundance and easy accessibility in the hairy skin, might be a good candidate for elderly patients.

One critical limitation of EPI‐NCSC therapy for cerebral ischemia is lack of sufficient information regarding their mechanism of action. However, potential mechanisms might be growth factor secretion, synaptogenesis, angiogenesis, neurogenesis, normalizing metabolic/microenvironmental profiles, enhanced autophagy, reduced scar thickness, immunomodulation, neural circuit reconstruction, apoptosis inhibition, and possibly replacing damaged cells.^7^, ^57^, ^58^ Nevertheless, further investigations are required to clarify the exact mechanism involved. Additionally, therapeutic effects of EPI‐NCSCs should be assessed in aging rats to mimic the conditions of elderly stroke patients.

## 5 CONCLUSION

In summary, the present findings suggest the therapeutic potential of EPI‐NCSCs in a rat model of ischemic stroke induced by middle cerebral artery occlusion. We also found that administration of EPI‐NCSCs via IA or IV routes immediately after reperfusion had created a comparable outcome to MSC‐IA, 7 days after transplantation.

## CONFLICT OF INTEREST

The authors declare that they have no competing interests.

## Supporting information

Table S1

Table S2
