# Supplementary material for: Epidermal neural crest stem cell transplantation as a promising therapeutic strategy for ischemic stroke
Source: CNS Neurosci Ther. 2020 Apr 12;26(7):670–81. doi: 10.1111/cns.13370 (PMC7298983; doi:10.1111/cns.13370)
Supplement: Supplementary file 1 — Table S1 [file CNS-26-670-s001.docx]

| **Animal model** | **Type of ischemia** | **Type of stem cell** | **Root of administration** | **Time of transplantation after stroke** | **Number of transplanted cells** | **Behavioral test (time of evaluation)** | **Main findings** | **Reference** |
| --- | --- | --- | --- | --- | --- | --- | --- | --- |
| Adult male Sprague-Dawley rats | MCAO  120 min | Rat BM-MSCs | IV | Immediately | 5×10^5^ | Modified neurologic severity score (1 and 4 weeks) | BM-MSCs improved functional recovery only 4 weeks after transplantation | (Goldmacher et al., 2013) |
| Adult female Wistar rats | MCAO  120 min | Rat BM-MSCs | IV | 1 day | 3×10^6^ | Rotarod test (1, 7 and 14 days) | BM-MSCs improved functional recovery only 14 days after transplantation | (Chen et al., 2003) |
| Adult male Wistar rats | MCAO  120 min | Rat BM-MSCs | IV | 1 day | 1×10^6^  Or  3×10^6^ | Modified neurologic severity score and rotarod tests (1, 7 and 14 days) | Only high dose of BM-MSCs and just based on the mNSS test improved functional recovery 14 days after transplantation | (Chen et al., 2001) |
| Adult male Sprague-Dawley rats | MCAO  120 min | Rat BM-MSCs | IV | 1 day | 3×10^6^ | Modified neurologic severity score (1 and 14 days) | BM-MSCs improved functional recovery 14 days after transplantation | (Liu et al., 2011) |
| Adult male Sprague Dawley rats | MCAO  120 min | Rat BM-MSCs | IV | 6 hours | 5×10^6^ | Modified neurologic severity score (1, 7, 14, 21 and 28 days) | BM-MSCs was not able to improve functional recovery | (Yang et al., 2014) |
| Adult male Sprague-Dawley | MCAO  90 min | Rat BM-MSCs | IV | 3 days | 2×10^6^ | Modified neurologic severity score (7 and 14 days) | BM-MSCs was not able to improve functional recovery | (Huang et al., 2017) |
| Adult male Wistar rats | Permanent focal cerebral ischemia | Rat BM-MSCs | IV | 1 day | 3×10^6^ | Modified neurologic severity score (1, 7, 14, 21 and 28 days) | BM-MSCs improved functional recovery starting at 2 weeks after transplantation | (Liu et al., 2010) |
| Adult female Sprague-Dawley rats | Permanent MCAO | Rat BM-MSCs | IV | 6 hours | 1×10^6^ | Limb Placement Test (1d, 14d, 35d) | BM-MSCs improved functional recovery starting at 14 days after transplantation | (Sasaki et al., 2016) |
| Adult male  Sprague–Dawley rats | Permanent MCAO | Rat BM-MSCs | IV | 1 day | 2×10^6^ | Modified neurologic severity score (1d, 7d, 14d, 28d) | BM-MSCs improved functional recovery starting at 14 days after transplantation | (Deng et al., 2010) |
| Adult male Sprague–Dawley rats | Permanent MCAO | Rat BM-MSCs | IV | 3 hours | 2×10^6^ | Modified neurologic severity score (1d, 7d, 14d, 28d) | BM-MSCs improved functional recovery starting at 14 days after transplantation | (Ye et al., 2013) |

Chen J, Li Y, Katakowski M, Chen X, Wang L, Lu D, et al. Intravenous bone marrow stromal cell therapy reduces apoptosis and promotes endogenous cell proliferation after stroke in female rat. Journal of neuroscience research 2003; 73: 778-786.

Chen J, Li Y, Wang L, Zhang Z, Lu D, Lu M, et al. Therapeutic benefit of intravenous administration of bone marrow stromal cells after cerebral ischemia in rats. Stroke 2001; 32: 1005-1011.

Deng YB, Ye WB, Hu ZZ, Yan Y, Wang Y, Takon BF, et al. Intravenously administered bmscs reduce neuronal apoptosis and promote neuronal proliferation through the release of vegf after stroke in rats. Neurological research 2010; 32: 148-156.

Goldmacher GV, Nasser R, Lee DY, Yigit S, Rosenwasser R, Iacovitti L. Tracking transplanted bone marrow stem cells and their effects in the rat mcao stroke model. PloS one 2013; 8.

Huang B, Jiang X-C, Zhang T-Y, Hu Y-L, Tabata Y, Chen Z, et al. Peptide modified mesenchymal stem cells as targeting delivery system transfected with mir-133b for the treatment of cerebral ischemia. International journal of pharmaceutics 2017; 531: 90-100.

Liu N, Zhang Y, Fan L, Yuan M, Du H, Cheng R, et al. Effects of transplantation with bone marrow-derived mesenchymal stem cells modified by survivin on experimental stroke in rats. Journal of translational medicine 2011; 9: 105.

Liu Z, Li Y, Zhang ZG, Cui X, Cui Y, Lu M, et al. Bone marrow stromal cells enhance inter-and intracortical axonal connections after ischemic stroke in adult rats. Journal of Cerebral Blood Flow & Metabolism 2010; 30: 1288-1295.

Sasaki Y, Sasaki M, Kataoka-Sasaki Y, Nakazaki M, Nagahama H, Suzuki J, et al. Synergic effects of rehabilitation and intravenous infusion of mesenchymal stem cells after stroke in rats. Physical therapy 2016; 96: 1791-1798.

Yang C, Liu H, Liu D. Mutant hypoxia‑inducible factor 1α modified bone marrow mesenchymal stem cells ameliorate cerebral ischemia. International journal of molecular medicine 2014; 34: 1622-1628.

Ye Z, Ye W, Deng Y, Wang J, Zhou G, Zhang X. Hif-1-modified bmscs improve migration and reduce neuronal apoptosis after stroke in rats. Chinese Science Bulletin 2013; 58: 3519-3528.
