# Supplementary material for: Epidermal neural crest stem cell transplantation as a promising therapeutic strategy for ischemic stroke
Source: CNS Neurosci Ther. 2020 Apr 12;26(7):670–81. doi: 10.1111/cns.13370 (PMC7298983; doi:10.1111/cns.13370)
Supplement: Supplementary file 2 — Table S2 [file CNS-26-670-s002.docx]

| **Animal model** | **Type of ischemia** | **Type of stem cell** | **Root of administration** | **Time of transplantation after stroke** | **Number of transplanted cells** | **Behavioral test (time of evaluation)** | **Main findings** | **Reference** |
| --- | --- | --- | --- | --- | --- | --- | --- | --- |
| Sprague Dawley rats | MCAO (60 min) | Rat BM-MSCs | IV | 1 day | Not defined | Modified neurologic severity score (7 days) | BM-MSCs improved functional recovery one week after transplantation | (Lu et al., 2016) |
| Adult male Sprague–  Dawley rats | MCAO (120 min) | Rat BM-MSCs | IV | 1 day | 2×106 | Neurologic severity score (7 days) | BM-MSCs improved functional recovery one week after transplantation | (Yu et al., 2012) |
| Adult male F344 Fisher rats | MCAO (90 min) | Rat BM-MSCs | IV | 3h | 3×106 | Modified neurologic severity score and rotarod tests (every day for 7 days) | BM-MSCs improved functional recovery from 4th to 7th day after transplantation | (Okazaki et al., 2008) |
| Adult female Wistar rats | MCAO (120 min) | Rat BM-MSCs | IV | 1 day | 1×106  or  3×106 | Modified neurologic severity score (1, 3 and 7 days) | BM-MSCs improved functional recovery from 3th to 7th day after transplantation | (Wu et al., 2007) |
| Adult male Sprague‐Dawley rats | MCAO (90 min) | Rat BM-MSCs | IV | 6h | 5×106 | Limb‐placement test (1, 3 and 7 days) | BM-MSCs improved functional recovery from 3th to 7th day after transplantation | (Chen et al., 2011) |
| Adult male Sprague‐Dawley rats | Permanent MCAO | Rat BM-MSCs | IV | 5 days | 5×106 | Modified neurologic severity score (1, 2 and 3 weeks) | BM-MSCs improved functional recovery one and two weeks after transplantation | (Ding et al., 2011) |
| Adult male Wistar rats | Embolic stroke | Rat BM-MSCs | IV | 1 day | 3×106 | Neurologic severity score (6h, 1, 2, 3 and 7 days) | BM-MSCs improved functional recovery from 3th to 7th day after transplantation | (Pirzad Jahromi et al., 2012) |
| Adult male Sprague‐Dawley rats | MCAO (120 min) | Rat BM-MSCs | IV | 1 day | 5×105 | Modified neurologic severity score (1, 7 and 28 days) | BM-MSCs improved functional recovery from 7th day after transplantation | (Yang et al., 2010) |
| Adult male  Sprague–Dawley rats | Permanent MCAO | Human BM-MSCs | IV | 3h | 1×107 | Limb placement test (3h, 1d, 3d, 4d, 7d) | BM-MSCs improved functional recovery from 3th day after transplantation | (Liu et al., 2006) |
| Adult male Sprague–Dawley rats | MCAO (120 min) | Human BM-MSCs | IV | 1 day | 2×106 | Modified neurologic severity score (3, 7 and 14 days) | BM-MSCs improved functional recovery from 3th to 7th day after transplantation | (Bang et al., 2012) |

Bang OY, Jin KS, Hwang MN, Kang HY, Kim BJ, Lee SJ, et al. The effect of cxcr4 overexpression on mesenchymal stem cell transplantation in ischemic stroke. Cell medicine 2012; 4: 65-76.

Chen C, Cheng Y, Chen J. Transfection of noggin in bone marrow stromal cells (bmscs) enhances bmsc‐induced functional outcome after stroke in rats. Journal of neuroscience research 2011; 89: 1194-1202.

Ding J, Cheng Y, Gao S, Chen J. Effects of nerve growth factor and noggin‐modified bone marrow stromal cells on stroke in rats. Journal of neuroscience research 2011; 89: 222-230.

Liu H, Honmou O, Harada K, Nakamura K, Houkin K, Hamada H, et al. Neuroprotection by plgf gene-modified human mesenchymal stem cells after cerebral ischaemia. Brain 2006; 129: 2734-2745.

Lu H, Liu X, Zhang N, Zhu X, Liang H, Sun L, et al. Neuroprotective effects of brain-derived neurotrophic factor and noggin-modified bone mesenchymal stem cells in focal cerebral ischemia in rats. Journal of Stroke and Cerebrovascular Diseases 2016; 25: 410-418.

Okazaki T, Magaki T, Takeda M, Kajiwara Y, Hanaya R, Sugiyama K, et al. Intravenous administration of bone marrow stromal cells increases survivin and bcl-2 protein expression and improves sensorimotor function following ischemia in rats. Neuroscience letters 2008; 430: 109-114.

Pirzad Jahromi G, Seidi S, Sadr S, Shabanzadeh A, Keshavarz M, Kaka G, et al. Therapeutic effects of a combinatorial treatment of simvastatin and bone marrow stromal cells on experimental embolic stroke. Basic & clinical pharmacology & toxicology 2012; 110: 487-493.

Wu J, Sun Z, Sun H-S, Wu J, Weisel RD, Keating A, et al. Intravenously administered bone marrow cells migrate to damaged brain tissue and improve neural function in ischemic rats. Cell transplantation 2007; 16: 993-1005.

Yang M, Wei X, Li J, Heine LA, Rosenwasser R, Iacovitti L. Changes in host blood factors and brain glia accompanying the functional recovery after systemic administration of bone marrow stem cells in ischemic stroke rats. Cell transplantation 2010; 19: 1073-1084.

Yu X, Chen D, Zhang Y, Wu X, Huang Z, Zhou H, et al. Overexpression of cxcr4 in mesenchymal stem cells promotes migration, neuroprotection and angiogenesis in a rat model of stroke. Journal of the neurological sciences 2012; 316: 141-149.
